# QTL Mapping and GWAS Reveal the Genetic Mechanism Controlling Soluble Solids Content in *Brassica napus* Shoots

**DOI:** 10.3390/foods10102400

**Published:** 2021-10-11

**Authors:** Xu Wu, Feng Chen, Xiaozhen Zhao, Chengke Pang, Rui Shi, Changle Liu, Chengming Sun, Wei Zhang, Xiaodong Wang, Jiefu Zhang

**Affiliations:** 1 School of Food and Biological Engineering, Jiangsu University, Zhenjiang 212013, China; xu_wu123@126.com (X.W.); liuchangle0323@163.com (C.L.); 2 Key Laboratory of Cotton and Rapeseed, Ministry of Agriculture and Rural Afairs, Institute of Industrial Crops, Jiangsu Academy of Agricultural Sciences, Nanjing 210014, China; minister119@126.com (F.C.); zxzhen2020@163.com (X.Z.); pangcke@163.com (C.P.); n77070299@163.com (R.S.); suncm8331537@gmail.com (C.S.); zhangwei1720@163.com (W.Z.); 3 State Key Laboratory of Crop Genetics and Germplasm Enhancement, Nanjing Agricultural University, Nanjing 210095, China

**Keywords:** *Brassica napus*, oilseed-vegetable-dual-purpose, shoot, soluble solids content, QTL mapping, GWAS, candidate genes

## Abstract

Oilseed-vegetable-dual-purpose (OVDP) rapeseed can effectively alleviate the land contradiction between crops and it supplements vegetable supplies in winter or spring. The soluble solids content (SSC) is an important index that is used to evaluate the quality and sugar content of fruits and vegetables. However, the genetic architecture underlying the SSC in *Brassica napus* shoots is still unclear. Here, quantitative trait loci (QTLs) for the SSC in *B. napus* shoots were investigated by performing linkage mapping using a recombinant inbred line population containing 189 lines. A germplasm set comprising 302 accessions was also used to conduct a genome-wide association study (GWAS). The QTL mapping revealed six QTLs located on chromosomes A01, A04, A08, and A09 in two experiments. Among them, two major QTLs, *qSSC/21GY.A04-1* and *qSSC/21NJ.A08-1*, accounted for 12.92% and 10.18% of the phenotypic variance, respectively. In addition, eight single-nucleotide polymorphisms with phenotypic variances between 5.62% and 10.18% were identified by the GWAS method. However, no locus was simultaneously identified by QTL mapping and GWAS. We identified AH174 (7.55 °Brix and 7.9 °Brix), L166 (8.9 °Brix and 8.38 °Brix), and L380 (8.9 °Brix and 7.74 °Brix) accessions can be used as superior parents. These results provide valuable information that increases our understanding of the genetic control of SSC and will facilitate the breeding of high-SSC *B. napus* shoots.

## 1. Introduction

Rapeseed (*Brassica napus*, AACC, 2n = 38) is a globally important oil crop with a large planting area and wide application [1]. The production of oilseed-vegetable-dual-purpose (OVDP) products is one of its typical uses. Stem-cutting once for use as a vegetable has no obvious effect on seed yield and has additional economic and ecological benefits [2]. The increase in the global population has meant that there is a great demand for edible oil and vegetables. Especially in winter and spring, crops grow slowly and green vegetables are in short supply. In addition, land resources available for vegetable and oilseed production are limited. The development of OVDP rapeseed can alleviate this contradiction to a certain extent [3]. Previous studies have suggested OVDP rapeseed varieties with high yields and quality will become the main objective of the market. However, previous reports on OVDP rapeseed mostly focused on yield and less on its quality.

*B. napus* shoots, as a fresh vegetable with rich nutrition, contain various vitamins, are rich in minerals, especially higher calcium, selenium, and zinc contents [2], and is a friendly vegetable to supplement selenium deficiency [4]. In addition, *B. napus* shoots fill the gap due to the lack of a seasonal vegetable supply, and also can be processed into dehydrated vegetables for long-term preservation [2]. Previous studies have suggested that *Brassica* vegetables improve human immunity, reduce the incidence of cardiovascular disease, and have the antioxidant capacity [5,6]. This means that *B. napus* shoots have considerable development potential. Bitter-tasting vegetables deter some people from choosing them because most consumers dislike bitter or strong-tasting vegetables and prefer milder and sweeter ones [7,8,9]. *Brassica* vegetables (e.g., Brussels sprouts, kale, mustard greens, broccoli, and red and white cabbage) contain bitter compounds, which include glucosinolates (GLSs). Their degradation products, such as isothiocyanates and thyroxine, contribute to the bitter and pungent taste [8,10,11], and limit consumer acceptance and intake of *Brassica* vegetables. However, some schemes that attempted to reduce bitterness by decreasing the content of bitter-tasting GLSs were not favored as several studies have demonstrated that GLSs and GLS degradation products intake have a cancer-inhibiting effect [12,13,14]. Thus, researchers believe that increasing sweetness is a positive predictor of acceptance and sugar is central because it can mask a taste perceived as bitter [15]. To date, there have been few reports about increasing the sugar content in *Brassica* vegetables, especially in *B. napus* shoots. Previous studies have shown that the soluble sugar content can be increased by adding plant nutrients rationally or by spraying sucrose onto the leaf surface to increase the sweetness of the plant [16,17]. Due to different environments and production cost limitations, these cultivation measures are not a total panacea. Therefore, we need to understand the genetic mechanism controlling sugar content in *B. napus* shoots and discover favorable alleles and their donor materials.

In fruits and vegetables, the soluble solids content (SSC) is used to represent sweetness. There are two main reasons for this. First, the SSC, as a flavor factor in fruit vegetables, contains most of the soluble sugars, but contains few organic acids and volatile aromas. The other reason is that the SSC can be quickly measured by a sugar refractometer, which has the advantages of being low-cost and simple for the operator, and is widely used to measure the sugar content in food. Numerous studies have shown that SSC affects the nutritional quality, sweetness and flavor of fruits and vegetables, such as tomatoes [18], muskmelon [19], white head cabbage (*Brassica oleracea* L.) [20], fresh-cut swede (*Brassica napus* L. var.) and turnip (*Brassica rapa* L. ssp.) [21]. According to previous statistics, cabbages have an average SSC of around 2–4 °Brix, rapeseeds have an average SSC around 4–6 °Brix, both lower than fruits such as melons (7.88–10.72 °Brix) and cherry tomatoes (5.0–8.5 °Brix). Thus, increasing the SSC can improve the quality and sweetness of fruits and vegetables. In addition, the SSC is a complex quantitative trait that is controlled by multiple genes and is susceptible to environmental conditions [22,23,24]. A common approach to understanding the genetic basis of complex traits is to identify associated quantitative trait loci (QTLs) [25]. Several researchers have used linkage analysis to map QTLs for SSCs in horticultural crops that use fruit as edible organs [26,27,28,29]. These crops include strawberry, peach, tomato, and yardlong bean. However, there have been few studies on leafy vegetables. Traditional QTL mapping in pedigrees is a powerful tool that allows researchers to understand the genetic mechanisms controlling complex traits [30]. In addition, the genome-wide association study (GWAS) is another important strategy used in quantitative trait research because it does not require population construction and it can detect the genetic architecture of traits, and can identify multiple related candidate genes. Recently, with the development of high-density single-nucleotide polymorphism (SNP) BeadCap and whole-genome sequencing technology, GWASs have been widely used to identify candidate genes that control quantitative traits in wheat [31], maize [32], and other crops. In *B. napus,* GWASs have been used in seed coat color [33], plant height, primary branch number [34], flowering time [35], disease [36], drought stress tolerance [37], and seed oil content [38]. In addition, the combined use of QTL mapping and GWASs has proved to be a useful tool when studying quantitative traits. At present, the combined strategy has been successfully applied to the analysis of quantitative traits in *B. napus*. For example, Kiran et al. [39] used a linkage mapping–GWAS joint strategy to investigate the architectural traits of rapeseed roots. The results revealed 11 QTL regions and 38 significant marker–trait associations were detected in the diversity set. In order to dissect the genetic architecture of rapeseed waterlogging tolerance, Wang et al. [30] employed QTL mapping and GWAS approaches to investigate seedling death rates using a recombinant inbred line (RIL) population and a GWAS population, and obtained 17 QTLs and 26 significant SNPs, respectively. Similarly, Sun et al. [40] aimed to identify and establish more reliable QTLs for seed oil content by combining QTL mapping and GWAS. Seven overlapping QTLs were identified by both methods in different environments. However, there have been few studies on the use of QTL mapping and GWAS for SSC in *B. napus* shoots, and the genetic mechanism controlling SSC is still not clear.

In this study, we mapped QTLs for SSC in *B. napus* shoots at the bolting stage using a RIL population containing 189 lines. This population was then used to construct a high-density SNP map [41]. To date, the RIL population has successfully been used to map QTLs for seed fatty acid composition [42], flowering time [43], and seed weight and shape [44]. In addition, a total of 302 diverse rapeseed accessions were subjected to GWAS to detect SNPs associate with SSC. Combining the QTL mapping and GWAS strategies provided insights that enhance our understanding of the genetic mechanism underlying SSC in *B. napus* shoots, and the results can be used to improve the sweetness of *Brassica* vegetables.

## 2. Materials and Methods

### 2.1. Plant Materials

The RIL population, named AH, contained 189 lines and was derived from a cross between “APL01” and “Holly” using the single seed descent method [41]. In this study, the population was planted under natural growing conditions in Nanjing, Jiangsu Province, China, and Guiyang, Guizhou Province, China, from September 2020 to March 2021 (referred to as 21 NJ and 21 GY, respectively). These two sites had different environmental conditions. A completely random block design was adopted and there were two replicates. The experimental unit was a two-row plot with 20 plants per row and 40 cm between the rows. Field management was carried out in accordance with local conventional methods.

The GWAS population was composed of 302 representative accessions and was selected from 520 natural population resources [33,45]. It was used to follow the genetic distance between SNPs (Table S1). These lines were assigned to three different germplasm types, namely spring (23 lines), winter (24), and semi-winter rapeseed (255). They mostly came from the world’s main rapeseed producing areas, including America (1), Australia (3), Canada (1), China (279), Denmark (1), France (1), Germany (8), Japan (3), Sweden (1), and South Korea (4), and were provided by the National Rape Engineering Research Center of Huazhong Agricultural University in Wuhan, Hubei Province, China. The population was used to perform the GWAS on SSC and was cultivated in Nanjing, Jiangsu Province for two consecutive years from September 2019 to March 2020 and from September 2020 to March 2021 (referred to as 20 NJ and 21 NJ, respectively). All planting rules and field management for this population followed the AH population. Additionally, the population has been widely used to analyze seed coat color [33], and the number of seeds per silique [45]. 

### 2.2. Measurement of SSC

At the bolting stage, *B. napus* shoots with a length of 20 cm, uniform thickness, complete leaf shape, no withering, no white heart, and no flower bud opening were chosen using the national agricultural industry-standard—“Grades and specifications of flowering Chinese cabbage” [46]. Three plants from each line were randomly selected, the leaves were removed, and the plant juice was measured. The upper, middle, and lower parts of the stem were selected to avoid SSC variations in different plant parts due to distinct metabolic levels. Each part was measured twice with a digital refractometer (PAL-1, Atago, Tokyo, Japan) with temperature compensation, and the SSC was expressed in °Brix. A total of 6 data were obtained. The mean of the six measurements represented the single plant material, and the mean phenotypic value of the three individual plants represented a particular line. Statistical analysis of the phenotypic data and calculation of broad-sense heritability were carried out by R [35]. The broad-sense heritability (*h*^2^) was calculated using the following formula: *h*^2^ = *σ_g_*^2^/(*σ_g_*^2^ + *σ_ge_*^2^/*n* + *σ_e_*^2^/*nr*) × 100%, where *σ_g_*^2^ is the genetic variance, *σ_ge_*^2^ is the interaction variance of the genotype with the environment, *σ_e_*^2^ is the error variance, *n* is the number of environments and *r* is the number of replications.

### 2.3. QTL Detection and GWAS of SSC

A high-density SNP map was constructed for the AH population. It covered 2027.53 cM with an average genetic distance of 0.72 cM between adjacent markers, and included 11,458 SNPs and 57 simple sequence repeats markers [41]. Windows QTL Cartographer 2.5 and the composite interval mapping model were used to determine the location and effects of the QTLs [47]. The logarithm of the odds (LOD) threshold was determined by 1000 permutation tests at *p* ≤ 0.05. Any QTLs with LOD values ≥ than the threshold were defined as a single QTL. The nomenclature for the QTLs was assigned according to Wang et al. [30]. The prefix “*qSSC*” before the slash indicates that the identified QTL was associated with SSC, the number following the slash represents the different environments, and the linkage group follows the decimal point. If more than one QTL was identified in a linkage group, then a number was added as a suffix (e.g., *qSSC/21NJ.A01-2*) [48].

The population structure, relative kinship, and linkage disequilibrium analysis were based on previous reports about the branch angle, plant height, and seed coat color [33,49,50]. In this study, we performed the GWAS using the Q + K model with a total of 19,167 high-quality SNPs. The Q + K matrix was used as covariables and we implemented the Mixed Linear Model (MLM) in TASSEL 5.1 [51]. A uniform Bonferroni threshold for the GWAS was set as –1og(1/n), where *n* is the total number of SNPs used in the association analysis. Here, –1og(1/19167) ≈ 4.28. When there are multiple significant SNPs in the 1 Mb interval and if two *r*^2^ ≥ 0.1, then these SNPs are classified as an association locus and are represented by the SNP with the minimum *p*-value. The Manhattan and QQ (Quantile-Quantile) plots were drawn by the R [35] package QQMAN [52].

### 2.4. Prediction of Candidate Genes

Sequences for predicted gene models at promising loci were retrieved from the *B. napus* “Darmor-*bzh*” Genome Browser (https://www.genoscope.cns.fr/brassicanapus/ (accessed on 15 April 2021)). Functional annotation of the genes was performed using a BLAST tool search and the TAIR database (https://www.arabidopsis.org/index.jsp (accessed on 15 April 2021)).

## 3. Results

### 3.1. Statistical Analysis of the SSC in the AH and GWAS Populations

**Figure 1:**
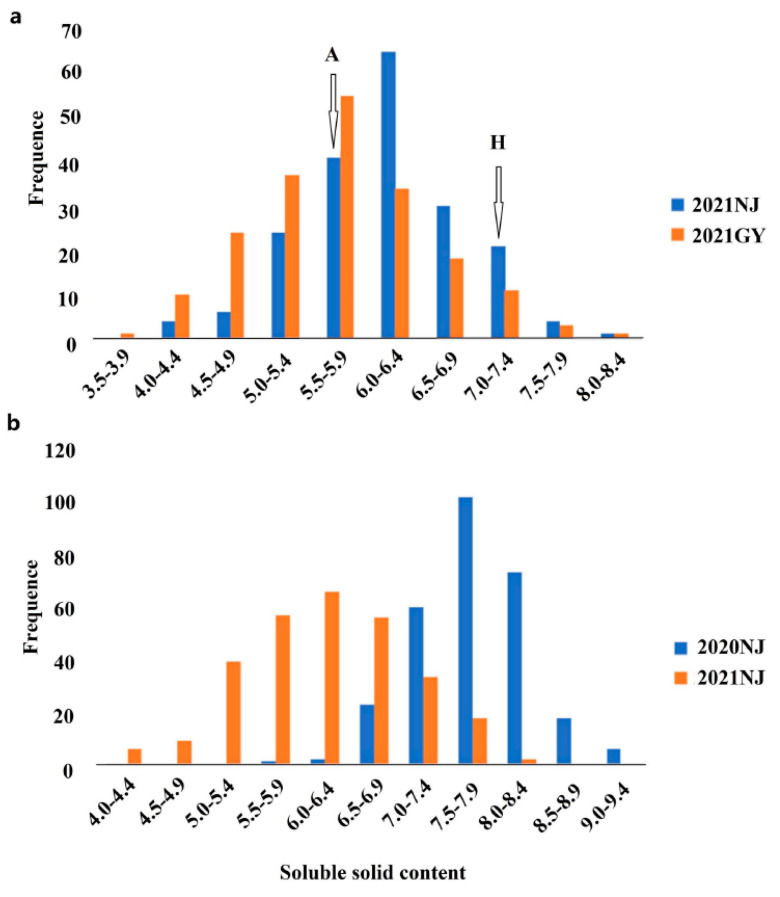
Phenotypic variation in SSC of the *B. napus* shoots. AH RIL (**a**) and GWAS populations (**b**). The *x*–axis represents the SSC (Brix), and the *y*–axis represents frequency. “A” and “H” indicate the average SSC of the female parent “APL01” and the male parent “Holly” in two experiments, respectively.

Phenotyping in the different environments was conducted to study the SSC distribution. The SSCs in both the AH and GWAS populations were normally distributed, suggesting that the trait had the characteristics of quantitative traits controlled by multiple genes, met the requirements of QTL mapping, and was suitable for genome-wide association analysis (Figure 1). In the AH population, the SSC ranged from 3.70 °Brix to 9.30 °Brix with coefficients of variation ranging from 11.5% to 14.3% between the two different sites (Table 1). The SSCs of the male parent “Holly” and the female parent “APL01” were 7.4 °Brix and 6.0 °Brix, in 21NJ, and 7.0 °Brix and 5.5 °Brix in 21 GY, respectively. There was a significant difference in SSC between the parents according to a *t* test (*p* < 0.01). Similarly, the SSC of the GWAS population ranged from 4.14 °Brix to 9.32 °Brix with coefficients of variation ranging from 7.5% to 12.8%. In addition, the *h*^2^ of the AH and GWAS populations was 52.7% and 62.1%, respectively, indicating that the trait was largely determined by genetic factors, but was also easily affected by environmental factors.

### 3.2. QTL Mapping of SSC

The SSCs of the AH population grown in the two environments were evaluated in the QTL analysis. A total of six QTLs were detected, which were located on chromosomes A01, A04, A08, and A09, and the phenotypic variation (PV) ranged from 5.58–12.92% (Table 2). Among them, two QTLs, *qSSC/21GY.A4-1* and *qSSC/21GY.A08-1* on A04 and A08, accounted for 12.92% and 10.18% of the PV in 21GY, respectively, and were considered to be two major QTLs. The other four QTLs accounted for 5.58–9.78% of the PV. In addition, four QTLs: *qSSC/21NJ.A01-2, qSSC/21NJ.A04-1, qSSC/21GY.A04-1,* and *qSSC/21GY.A08-1*, had negative additive effects of –0.22, –0.17, –0.74, and –0.52, respectively. This meant that the positive alleles for increasing SSC were inherited from “Holly”.

### 3.3. Genome-Wide Association Analysis of SSC

**Figure 2:**
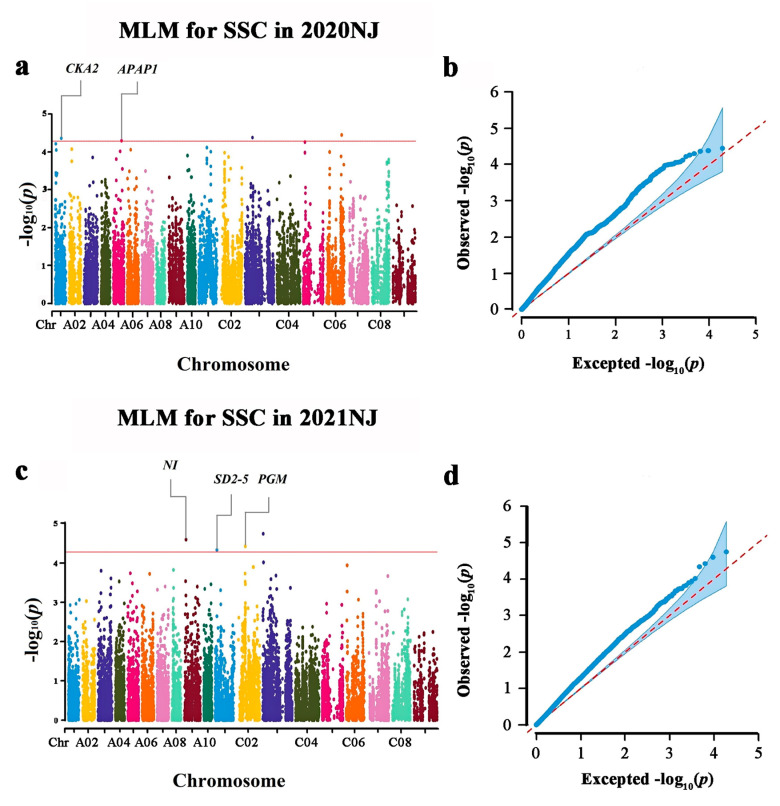
Genome-wide association study of *B. napus* shoots SSC. (**a**) Manhattan plot of the MLM for SSC in 2020 NJ; (**b**) Quantile–Quantile plot of the MLM for SSC; (**c**) Manhattan plot of the MLM for SSC in 2021 NJ; (**d**) Quantile–Quantile plot of the MLM for SSC. The red rings represent the suggestive threshold of −log10(*p*) = 4.28. GWAS loci are indicated above the threshold, and the corresponding genes are annotated. If more than one candidate gene was identified within the observed linkage disequilibrium decay of one locus, the most likely candidate gene is annotated in the Manhattan plot. Full gene information is provided in Table 4.

The results of the trait–SNP association analysis are shown in Figure 2 and Table 3. A total of eight significant SNPs were detected on chromosomes A01, A05, A09, C01, C02, C03, and C06 at *p* < 5.2 × 10^−5^ [*p* = 1/19167, and –log10(*p*) = 4.28] in the two experimental years. Among these, none of the loci was detected repeatedly in 2020 and 2021, indicating that the trait was susceptible to environmental factors. The most significant SNP (Bn-scaff_16614_1-p1120223) locus on C03 explained 10.18% of the PV in 21 NJ. The other seven SNPs explained 9.22–9.69% of the PV.

Combining the results of the QTL mapping and GWAS showed that there were a total of 14 loci for SSC, which were distributed on the A01 (3 loci), A04 (2), A05 (1), A08 (1), A09 (2), C01 (1), C02 (1), C03 (2), and C06 (1) chromosomes. Furthermore, chromosomes A01 and A09 were found in both populations. However, no locus was simultaneously detected by the QTL mapping and GWAS approaches.

### 3.4. Candidate Genes Prediction for SSC

The QTL mapping and GWAS approaches predicted seven candidate genes for the six loci involved in the SSC biosynthesis pathway (Table 4). *BnaA09g41790D* is an important gene located at 29.12–29.13 Mbp on chromosome A09. Its *Arabidopsis* homologous gene encodes the enzyme neutral invertase (*NI*), which is involved in sucrose metabolism and irreversibly dividing sucrose into glucose and fructose [53]. Similarly, the GWAS results showed that six candidate genes were annotated as being associated with the biological processes of carbohydrate synthesis, transport, and metabolism. *Bna-A09-g08200D* and *Bna-A09-g08760D*, found at 3 kb downstream and 378 kb upstream from Bn-A09-p3994437, respectively, were also located on chromosome A09, and their homologous genes in *Arabidopsis* are *MGD3* and *STP7*, respectively. *MGD3* encodes galactosyldiglyceryl esterase and its overexpression leads to a decrease in sucrose content in *Arabidopsis* stems [54]. *STP7* encodes monosaccharide transporters, which are members of the sugar transporter family that are involved in sugar uptake in cells. They are also involved in cell wall sugar uptake and recycling [55,56]. Another important candidate gene, *BnaC02g20320D*, was located 2 kb upstream from Bn-Scaff-17566_1-P21523 on chromosome C02. The glucose–phosphate transgenic enzyme *PGM*, encoded by the *Arabidopsis* homologous gene *AT1G70820*, can catalyze the reversible transformation of α-D-glucose-l-phosphate (G-1-P) and α-D-glucose-6-phosphate (G-6-P). During photosynthesis, *PGM* proceeds along the direction of G-1-P formation, synthesizing sucrose in the cytosol and starch in the plastid [57]. In addition, three candidate genes were distributed on A01, A05, and C01, respectively. Their *Arabidopsis* homologous genes are involved in cell cycle control and cell wall activities. For example, *APAP1* and *SD2-5* are associated with the synthesis of cell wall polysaccharides, and *CKA2* is involved in the control of the cell cycle, delaying flowering time, and facilitating the accumulation of photosynthate. Whether the above genes control the accumulation of soluble solids still needs further functional verification.

### 3.5. Selection of Superior Parents 

The statistical results were used to select the top ten accessions with the highest SSCs from the different environments (Table 5). In the AH population, the ten lines with the highest SSC ranged from 7.3–8.32 °Brix with a mean of 7.54 °Brix in 21 NJ, and 6.8–9.3 °Brix with a mean of 7.6 °Brix in 21 GY. The phenotypic values for AH174 were 7.55 °Brix and 7.9 °Brix for the two environments, respectively, and it was found simultaneously in both environments. Similarly, in the GWAS population, the ten lines with the highest SSCs had values that ranged from 8.90–9.32 °Brix with a mean of 9.06 °Brix in 20 NJ, and 7.74–8.38 °Brix with a mean of 7.93 °Brix in 21 GY. Meanwhile, the phenotypic values for L166 and L380 were both 8.90 °Brix in 20 NJ, and 8.38 °Brix and 7.74 °Brix in 21 NJ for the two experimental years, respectively. Additionally, the AH174, L166, and L380 accessions were all late-bolting cultivars. This suggested that late varieties had longer photosynthesis periods, which would improve the accumulation of sucrose. In summary, the AH174, L166, and L380 accessions performed steadily and could be considered as donor parental materials.

## 4. Discussion

Rapeseed is a globally important oil crop and occupies a large amount of cultivated land across the world. The popularization of the OVDP rapeseed varieties effectively alleviates land resource tension. In order to ensure that stem-cutting once does not affect rapeseed yields, but allows a harvest of *B. napus* shoots as fresh vegetables, scientists have bred many new varieties, which not only meet the edible oil demand, but also fill the gap due to the lack of a seasonal vegetable supply, which maximizes the benefits of rapeseed. The *B. napu*s shoots can be processed into dehydrated vegetables, which is convenient for long-term preservation, to extend the store season, convenient for transportation and food, with high added value. In addition, compared to traditional shoots (e.g., red and white cabbage), they have a higher nutritional quality, especially higher calcium, selenium, and zinc contents [2]. Nevertheless, flavor and texture are important quality characteristics of fruits and vegetables and are major factors affecting the sensory perception and consumer acceptance of foods [58]. At present, the genetic mechanism controlling its quality traits remains unknown so future research needs to concentrate on identifying this mechanism and improving the quality of *B. napus* shoots. Most previous reports on SSC concentrated on crops that use fruit as the harvesting organ and their sugar accumulation is complete when the fruits are ripe, such as tomatoes, peaches, and apples. In contrast, the sugar content in *B. napus* reaches its peak at the bolting stage, which is also the best time for cutting. On this basis, we investigated the SSC of *B. napus* shoots at the bolting stage. In order to reduce damage to the original flavor of *B. napus* shoots, we adopted the rapid measurement of saccharinity method with a refractometer in this experiment. The results showed that this method was efficient, feasible, and convenient.

Based on the phenotypic data, we calculated mean SSC values of 5.89 °Brix and 6.98 °Brix for the AH and GWAS populations, respectively, and compared them with other crops. Natalia et al. [59] measured the average SSC of the external, middle and internal parts of the celery petioles with a refractometer and ranged from 3.49 °Brix to 3.74 °Brix with an average of 3.61 °Brix; Kramchote et al. [60] measured the SSC ranging from 3.45 °Brix to 5.23 °Brix with a mean of about 4.34 °Brix in 135 cabbage samples; the SSC of 126 cherry tomatoes was measured by Sun et al. [61] and ranged from 5.0–8.5 °Brix with a mean of 6.6 °Brix; and Li et al. [62] measured the SSC of stylar-end and equatorial locations of 360 melons, with average values ranging from 7.88 °Brix to 10.72 °Brix. It can be seen that the average SSC in *B. napus* shoots was higher than that of celery and cabbage, lower than that of melon, and approximately equal to the cherry tomato. The high SSC in *B. napus* shoots indicates that they have important application potential as fresh vegetables. Furthermore, among the ten selected accessions with the highest SSCs in this study, the average SSC in the AH and GWAS populations ranged from 7.54 °Brix to 9.06 °Brix. These results are higher than those for cherry tomato and near the mean SSC for melon. 

Six QTLs associated with SSC in the AH population were detected, with PVs ranging from 5.58% to 12.92%. No overlapping loci were found. There are two possible reasons for this. First, the genetic effects associated with SSC are mild and second, the environment can influence QTL expression and its magnitude because the environment represents the manifestation of complex biotic, abiotic, and agronomic factors [63]. Indeed, in this study, environmental factors were hard to control under field conditions. A large population, a high-density genetic map and replicated experiments in multiple environments are considered as three key factors for increasing statistical power and precision in detecting QTLs [64]. Thus, this study can be improved by conducting multi-locus experiments over different years to ensure that stable expressions of the major genes are obtained. In addition, all the QTLs were distributed in the A subgenome. *B. napus* (AACC, 2n = 38) is an allotetraploid derived from the hybridization of its diploid progenitor species *B. rapa* (AA, 2n = 20) and *B. oleracea* (CC, 2n = 18), and has naturally undergone polyploidization. Thus, the chromosomes in the *B. napus* genome are highly homologous to the chromosomes in *B. oleracea* and *B. rapa*. Future research may find more relationships among them or other *Brassica* vegetables (e.g., kale, mustard greens, and red and white cabbage).

To reduce the risk of false positives, researchers generally prefer using the stringent mixed model in a GWAS, which accounts for kinship and structure, to identify the association signals. Furthermore, the MLM model is considered to be more suitable than the general linear model when conducting a GWAS. The results obtained by the MLM are more accurate, and this has been confirmed by several studies [65,66]. In this study, eight SNPs were identified by the MLM model and their PVs ranged from 9.22% to 10.18%. However, all the SNPs were only detected in one environment, indicating that the trait was susceptible to environmental influence. Comparing the results of QTL mapping and the GWAS showed that A01 and A09 shared the same chromosomes in the two populations. However, no locus could be simultaneously detected by both approaches. There have been similar cases in previous studies. For example, Wang et al. [30] used the same populations to identify loci for waterlogging tolerance and no overlapping loci were detected by both the QTL mapping and the GWAS. Tarka et al. [67] detected a major QTL for wing length in great reed warblers, but when a GWAS approach was performed, no distinct associations were identified [68]. Yabe et al. [69] used the GWAS and QTL mapping to investigate the traits associated with buckwheat yield and few common loci were detected by the two approaches. The disagreement between the GWAS and QTL mapping is partly explained by the difference in population structures and epistasis. According to Hansson et al. [68], the main reason for the different results obtained by the two methods is the conceptual differences in the genotypic association with PV. The QTL mapping method exploits recent recombination events in the pedigree, while the GWAS takes advantage of historical recombination events in large populations. In general, combining the GWAS and QTL mapping can alleviate the limitations of each approach and improve the reliability of the positioning results. Furthermore, the combined strategy can be used to identify other quality traits for *B. napus* shoots and further gene mining would contribute to the breeding of superior *B. napus* shoots. In this study, the combination of GWAS and QTL mapping strategies was merely used to preliminarily locate SSC in *B. napus*. The QTLs and SNPs associated with SSC obtained in the present study were not compared with other studies due to the lack of related research. Thus, multi-locus experiments over different years and the validation of candidate genes function should be conducted in the subsequent studies.

Despite the large number of mapping publications on crop SSCs, few genes have an established role in *B. napus* shoot sweetness regulation, which greatly hampers the progress of candidate gene mining. However, this study has identified two important candidate genes, *BnaA09g41790D* and *BnaC02g20320D*, whose *Arabidopsis* homologous genes are *NI* and *PGM*, respectively. Sucrose is the chief contributor to sweetness. Sucrose phosphate synthase, sucrose synthase, and *NI* are the three important enzymes involved in sucrose synthesis and cleavage in higher plants [70,71,72]. Neutral invertase activity is more intense during plant growth and the immature stages, and it regulates sucrose level by decomposing sucrose into fructose and glucose in plants [72]. In contrast, *PGM* regulates starch levels in fruit and catalyzes the reversible reaction of G-1-P to G-6-P. It can be converted into G-1-P for the synthesis of starch or sucrose during photosynthesis. Inhibition of *PGM* activity significantly impedes plant photosynthesis and reduces the starch and sucrose contents [73]. Additionally, we also identified candidate genes involved in sugar transport, cell wall synthesis, and cell cycle regulation in the vicinity of the significant SNPs (Table 4), but these functions need to be further verified. The candidate genes and their associated loci detected here improve our understanding of the genetic mechanism controlling SSC. In addition, the results of this study may facilitate the breeding of *B. napus* shoots and other *Brassica* vegetables with high sweetness in the future.

## 5. Conclusions

*B. napus* shoots, as a fresh vegetable with rich nutrition, have great prospects in the processing and application of fruit and vegetable by-products. In the present study, the SSC was investigated to represent sweetness in *B. napus*. The combination of QTL mapping and GWAS was used to decipher the genetic architecture underlying sweetness. QTL mapping revealed six QTLs for SSC, and two major QTLs *qSSC/21GY.A4-1* and *qSSC/21GY.A08-1* accounted for 12.92% and 10.18% of the PV, respectively. Meanwhile, eight significant SNPs associated with SSC were obtained by GWAS. In addition, we identified three lines (AH174, L166 and L380) that can be used as superior parents in breeding programs. Our research provides valuable information for better understanding the genetic control of sweetness in *B. napus*.

## Figures and Tables

**Table 1:** Statistical analysis of soluble solids content in *B. napus* shoots.

Trait	Population	Environment	Mean ± SD	Mode (°Brix)	Min (°Brix)	Max (°Brix)	Variable Coefficient/CV(%) ^1^	Broad-Sense Heritability/*h*^2^(%) ^2^
Soluble solids content	Recombinant inbred linepopulation	21 Nanjing	6.10 ± 0.70	6.27	4.07	8.32	11.5	52.7
		21 Guiyang	5.68 ± 0.81	5.90	3.70	9.30	14.3	
	GWASpopulation	20 Nanjing	7.73 ± 0.58	7.50	5.90	9.32	7.50	61.1
		21 Nanjing	6.24 ± 0.80	6.56	4.14	8.38	12.80	

^1^ CV is an abbreviation of the coefficient of variation, which was estimated as the ratio of the standard deviation to the mean of all accessions. ^2^ *h*^2^ is broad-sense heritability; *h*^2^ = *σ_g_*^2^/(*σ_g_*^2^ + *σ_ge_*^2^/*n* + *σ_e_*^2^*/nr*) × 100%, where *σ_g_*^2^ is the genetic variance, *σ_ge_*^2^ is the variance due to the genotype × environment interaction, *σ_e_*^2^ represents the residual error, *n* is the number of environments (years), and *r* is number of replicates.

**Table 2:** Quantitative trait loci for soluble solids content of the recombinant inbred line population under different environmental conditions.

Population	QTL	Chromosome	Position	LOD ^1^	Additive Effect ^2^	*R*^2^ (%) ^3^	SNP Interval	Physical Position (bp)	Environment
Recombinant inbred line population	*qSSC/21NJ.A01-1*	A01	85.41	3.1	0.19	5.98	Bn-A01-p21532512-BnGMS33	18,199,676–18,199,725	21 NJ
	*qSSC/21NJ.A01-2*	A01	104.51	4.0	−0.22	8.19	Bn-scaff_15879_1-p327427-Bn-scaff_15879_1-p715384	31,567,749–31,567,806	21 NJ
	*qSSC/21NJ.A04-1*	A04	70.01	2.9	−0.17	5.58	Bn-A04-p17742799-Bn-A04-p18125679	18,471,893–18,471,942	21 NJ
	*qSSC/21NJ.A09-1*	A09	72.71	5.0	0.22	9.78	Bn-scaff_16361_1-p1749058-Bn-A09-p28925363	29,297,004–29,298,009	21 NJ
	*qSSC/21GY.A04-1*	A04	4.11	3.9	−0.74	12.92	Bn-A04-p1274596-Bn-A04-p1900422	1,640,081–1,640,130	21 GY
	*qSSC/21GY.A08-1*	A08	53.31	3.0	−0.52	10.18	Bn-A08-p18592850-Bn-A08-p19573947	16,352,113–16,352,162	21 GY

^1^ LOD, logarithm of the odds; ^2^ additive effects is the accumulation of the genotype values of multiple micro-effect genes that affect quantitative traits, also known as the breeding value of a trait, and are the main component of the phenotypic value of a trait; ^3^ *R*^2^, proportion of phenotypic variation explained by the detected QTLs.

**Table 3:** Significant genome-wide association study single-nucleotide polymorphisms of soluble solids content in mixed linear model.

Marker	Chromosome	Position (bp)	−lg(*p*) ^1^	*R*^2^(%) ^2^	Environment
Bn-A01-p15502589	A01	12943376	4.36	9.39	20 NJ
Bn-A05-p19436126	A05	17655794	4.30	9.22	20 NJ
Bn-A09-p3994437	A09	3999318	4.60	9.69	21 NJ
Bn-scaff_15838_5-p335984	C01	3176216	4.33	9.27	21 NJ
Bn-scaff_17566_1-p21523	C02	16722668	4.42	9.48	21 NJ
Bn-scaff_16614_1-p1120223	C03	971293	4.75	10.18	21 NJ
Bn-scaff_16352_1-p308563	C03	15443140	4.38	9.36	20 NJ
Bn-scaff_16874_1-p411591	C06	31817621	4.45	9.61	20 NJ

^1^ −lg(*p*) represents a uniform Bonferroni threshold for the genome-wide association study. ^2^ *R*^2^, proportion of phenotypic variation explained by the detected single-nucleotide polymorphisms.

**Table 4:** Candidate gene information of soluble solids content associated loci in *B. napus* shoots.

Marker	Rapeseed Gene	Physical Position	Homologs in *A. thaliana*	Functional Annotation
QTL region				
Bn-scaff_16361_1-p1749058-Bn-A09-p28925363	*BnaA09g41790D*	A09:29,129,265–29,131,192	AT4G34860	plant neutral invertase family protein (*NI*)
GWAS region				
Bn-A01-p15502589	*BnaA01g21040D*	A01:12,943,079–12,945,493	AT3G50000	casein kinase II, alpha chain 2 (*CKA2*)
Bn-A05-p19436126	*BnaA05g23350D*	A05:17,683,715–17,684,062	AT3G45230	hydroxyproline-rich glycoprotein family protein (*APAP1*)
Bn-A09-p3994437	*BnaA09g08200D*	A09:3,996,167–3,999,048	AT2G11810	glycolipid biosynthetic process (*MGD3*)
Bn-scaff_15838_5-p335984	*BnaA09g08760D*	A09:4,377,564–4,379,821	AT4G02050	sugar transporter protein 7 (*STP7*)
	*BnaC01g06180D*	C01:3,255,144–3,256,301	AT4G32300	S-domain-2 5 (*SD2-5*)
Bn-scaff_17566_1-p21523	*BnaC02g20320D*	C02:16,723,975–16,724,687	AT1G70820	Phosphoglucomutase (*PGM*)

**Table 5:** The top ten of *B. napu*s shoots varieties with the highest soluble solids content in different environments.

Population	Environment	Material Name	Soluble Solid Content (°Brix)	Average
Recombinant inbred line population	21 NJ	AH124	8.32	7.54
		AH194	7.75	
		AH174	7.55	
		AH120	7.50	
		AH162	7.45	
		AH083	7.43	
		AH193	7.42	
		AH192	7.35	
		AH117	7.30	
		AH195	7.30	
	21 GY	AH169	9.30	7.60
		AH173	8.10	
		AH174	7.90	
		AH164	7.80	
		AH166	7.40	
		AH180	7.40	
		AH042	7.36	
		AH188	7.00	
		AH029	6.90	
		AH121	6.80	
GWAS population	20 NJ	L452	9.32	9.06
		L192	9.30	
		L449	9.20	
		L456	9.10	
		L247	9.00	
		L360	9.00	
		L465	9.00	
		L145	8.90	
		L166	8.90	
		L380	8.90	
	21 NJ	L166	8.38	7.93
		L527	8.12	
		L523	7.98	
		L275	7.92	
		L363	7.90	
		L381	7.86	
		L260	7.86	
		L510	7.76	
		L392	7.76	
		L380	7.74	

## Data Availability

All data are reported in this manuscript.

## Supplementary Materials

The following are available online at https://www.mdpi.com/article/10.3390/foods10102400/s1, Table S1: Information of the 302 association panel and group information based on population structure.

## Abbreviations

OVDP: oilseed-vegetable-dual-purpose; *B. napus*: *Brassica napus*; SSC: soluble solid content; QTL: quantitative trait locus; GWAS: genome-wide association Study; RIL: recombinant inbred line; GLSs: glucosinolates; MLM: Mixed Linear Model; PV: phenotypic variation; *NI*: neutral invertase; G-1-P: α-D-glucose-l-phosphate; G-6-P: α-D-glucose-6-phosphate; SNP: single-nucleotide polymorphism.

## References

[1] Hasan K., Tamanna N., Haque M.A. (2017). Biochemical and histopathological profiling of Wistar rats treated with rapeseed (*Brassica napus*) oil. Food Sci. Hum. Well..

[2] Huang H., Shi Y., Liu T., Zhou Y. (2014). Oilseed-vegetable-dual-purpose rape key technology research and its application prospect analysis. Agric. Sci..

[3] Wang Y., Wang G. (2007). Research status and development countermeasures of dual-use rape (in Chinese with an English abstract). J. Anhui Agric. Sci..

[4] Wang H. (2018). New-demand oriented oilseed rape industry developing strategy (in Chinese with an English abstract). Chin. J. Oil Crops Sci..

[5] Mandrich L., Caputo E. (2020). Brassicaceae-derived anticancer agents: Towards a green approach to beat cancer. Nutrients.

[6] Podsdek A. (2007). Natural antioxidants and antioxidant capacity of *Brassica* vegetables: A review. LWT-Food Sci. Technol..

[7] Doorn H.V., Van D., Holst G.V., Raaijmakers-Ruijs N., Postma E., Groeneweg B., Jongen W. (1998). The glucosinolates sinigrin and progoitrin are important determinants for taste preference and bitterness of Brussels sprouts. J. Sci. Food Agric..

[8] Drewnowski A., Gomez-Carneros C. (2001). Bitter taste, phytonutrients, and the consumer: A review. Am. J. Clin. Nutr..

[9] Dinehart M.E., Hayes J.E., Bartoshuk L.M., Lanier S.L., Duffy V.B. (2006). Bitter taste markers explain variability in vegetable sweetness, bitterness, and intake. Physiol. Behav..

[10] Fenwick G.R., Griffiths N.M., Heaney R.K. (1983). Bitterness in brussels sprouts (*Brassica oleracea* L. var. gemmifera): The role of glucosinolates and their breakdown products. J. Sci. Food Agric..

[11] Engel E., Baty C., Le Corre D., Souchon I., Martin N. (2002). Flavor-active compounds potentially implicated in cooked cauliflower acceptance. J. Agric. Food Chem..

[12] Poppel G.V., Verhoeven D.T., Verhagen H., Goldbohm R.A. (1999). *Brassica* vegetables and cancer prevention. Epidemiology and mechanisms. Adv. Exp. Med. Biol..

[13] Bazzano L.A., Jiang H., Ogden L.G., Loria C.M., Suma V., Leann M., Whelton P.K. (2002). Fruit and vegetable intake and risk of cardiovascular disease in US adults: The first national health and nutrition examination survey epidemiologic follow-up study. Am. J. Clin. Nutr..

[14] Verkerk R., Schreiner M., Krumbein A., Ciska E., Dekker M. (2009). Glucosinolates in *Brassica* vegetables: The influence of the food supply chain on intake, bioavailability and human health. Mol. Nutr. Food Res..

[15] Beck T.K., Jensen S., Bjoern G.K., Kidmose U. (2014). The masking effect of sucrose on perception of bitter compounds in *Brassica* vegetables. J. Sens. Stud..

[16] Suwanarit A., Sestapukdee M. (1989). Stimulating effects of foliar k-fertilizer applied at the appropriate stage of development of maize: A new way to increase yield and improve quality. Plant Soil.

[17] Gene E., Lester J.L., Jifon D.J., Makus (2010). Impact of potassium nutrition on postharvest fruit quality: Melon (*Cucumis melo* L) case study. Plant Soil.

[18] Beckles D.M. (2012). Factors affecting the postharvest soluble solids and sugar content of tomato (*Solanum lycopersicum* L.) fruit-ScienceDirect. Postharvest Biol. Technol..

[19] Mata M., José N., Natera M., Rafael J. (2009). Effect of growth regulators on the epicarp, mesocarp and total solublesolids of muskmelon (*Cucumis melo* L.) fruit cv. Edisto 47. Rev. Cient. UDO Agric..

[20] Viskeliene A., Samuoliene G., Karkleliene R., Viskelis P., Sasnauskas A., Duchovskis P. (2017). Quality and developmental changes in white head cabbage (*Brassica oleracea* L.) and radish (*Raphanus sativus* L.) during winter storage. Zemdirbyste.

[21] Helland H.S., Leufvén A., Bengtsson G.B., Skaret J., Wold A.B. (2016). Postharvest biology and technology. Postharvest Biol. Technol..

[22] Fridman E., Liu Y., Carmel-Goren L., Gur A., Shoresh M., Pleban T., Eshed Y., Zamir D. (2002). Two tightly linked QTLs modify tomato sugar content via different physiological pathways. Mol. Genet. Genom..

[23] Mallor C., Balcells M., Mallor F., Sales E. (2011). Genetic variation for bulb size, soluble solids content and pungency in the Spanish sweet onion variety Fuentes de Ebro. Response to selection for low pungency. Plant Breed..

[24] Samykanno K., Pang E., Marriott P.J. (2013). Genotypic and environmental effects on flavor attributes of ‘Albion’ and ‘Juliette’ strawberry fruits. Sci. Hortic.-Amst..

[25] Tecle I.Y., Menda N., Buels R.M., Knaap E., Mueller L.A. (2010). solQTL: A tool for QTL analysis, visualization and linking to genomes at SGN database. BMC Bioinform..

[26] Etienne C., Rothan C., Moing A., Plomion C., Bodénès C., Svanella-Dumas L., Cosson P., Pronier V., Monet R., Dirlewanger E. (2002). Candidate genes and QTLs for sugar and organic acid content in peach [*Prunus persica* (L.) Batsch]. Theor. Appl. Genet..

[27] Paterson A.H., Damon S., Hewitt J.D., Zamir D., Tanksley S.D. (1991). Mendelian factors underlying quantitative traits in tomato: Comparison across species, generations, and environments. Genetics.

[28] Kongjaimun A., Somta P., Tomooka N., Kaga A., Vaughan D.A., Srinives P. (2013). QTL mapping of pod tenderness and total soluble solid in yardlong bean [*Vigna unguiculata* (L.) Walp. subsp. unguiculata cv.-gr. sesquipedalis]. Euphytica.

[29] Castro P., Lewers K.S. (2016). Identification of quantitative trait loci (QTL) for fruit-quality traits and number of weeks of flowering in the cultivated strawberry. Mol. Breed..

[30] Wang X., Sun L., Li W., Peng M., Chen F., Zhang W., Sun C., Chen S., Hua W., Zhang J. (2020). Dissecting the genetic mechanisms of waterlogging tolerance in *Brassica napus* through linkage mapping and a genome-wide association study. Ind. Crops Prod..

[31] Afzal F., Li H., Gul A., Subhani A., Ali A., Mujeeb-Kazi A., Ogbonnaya F., Trethowan R., Xia X., He Z. (2019). Genome-wide analyses reveal footprints of divergent selection and drought adaptive traits in synthetic-derived wheats. G3 (Bethesda).

[32] Samayoa L., Malvar R., Olukolu B.A., Holland J.B., Butrón A. (2015). Genome-wide association study reveals a set of genes associated with resistance to the mediterranean corn borer (*Sesamia nonagrioides* L.) in a maize diversity panel. BMC Plant Biol..

[33] Wang J., Xian X., Xu X., Qu C., Liu L. (2017). Genome-wide association mapping of seed coat color in *Brassica napus*. J. Agric. Food Chem..

[34] Li F., Chen B., Xu K., Yan G., Qiao J., Li J., Li H., Li L., Xiao X., Zhang T. (2016). A genome-wide association study of plant height and primary branch number in rapeseed (*Brassica napus*). Plant Sci..

[35] Xu L., Hu K., Zhang Z., Guan C., Chen S., Wei H., Li J., Wen J., Yi B., Shen J. (2015). Genome-wide association study reveals the genetic architecture of flowering time in rapeseed (*Brassica napus* L.). DNA Res..

[36] Fikere M., Barbulescu D.M., Malmberg M.M., Spangenberg G.C., Cogan N.O.I., Daetwyler H.D. (2020). Meta-analysis of GWAS in canola blackleg (*Leptosphaeria maculans*) disease traits demonstrates increased power from imputed whole-genome sequence. Sci. Rep..

[37] Shahzad A., Qian M., Sun B., Mahmood U., Lu K. (2021). Genome-wide association study identifies novel loci and candidate genes for drought stress tolerance in rapeseed. Oil Crop Sci..

[38] Pal L., Sandhu S.K., Bhatia D. (2021). Genome-wide association study and identification of candidate genes for seed oil content in *Brassica napus*. Euphytica.

[39] Kiran A., Wakeel A., Snowdon R., Friedt W., Léon J. (2019). Genetic dissection of root architectural traits by QTL and genome-wide association mapping in rapeseed (*Brassica napus*). Plant Breed..

[40] Sun F., Liu J., Hua W., Sun X., Wang X., Wang H. (2016). Identification of stable QTLs for seed oil content by combined linkage and association mapping in *Brassica napus*. Plant Sci..

[41] Wang X., Yu K., Li H., Qi P., Feng C., Zhang W., Chen S., Hu M., Zhang J. (2015). High-density SNP map construction and QTL identification for the apetalous character in *Brassica napus* L.. Front. Plant Sci..

[42] Chen F., Zhang W., Yu K., Sun L., Gao J., Zhou X., Peng Q., Fu S., Hu M., Long W. (2018). Unconditional and conditional QTL analyses of seed fatty acid composition in *Brassica napus* L.. BMC Plant Biol..

[43] Yu K., Wang X., Li W., Sun L., Peng Q., Chen F., Zhang W., Guan R., Zhang J. (2019). Identification and physical mapping of QTLs associated with flowering time in *Brassica napus* L.. Euphytica.

[44] Sun L.J., Wang X., Yu K., Li W., Peng Q., Chen F., Zhang W., Fu S., Xiong D., Chu P. (2018). Mapping of QTLs controlling seed weight and seed-shape traits in *Brassica napus* L. using a high-density SNP map. Euphytica.

[45] Sun C., Chen F., Chen S., Peng Q., Zhang W., Yi B., Zhang J., Fu T. (2020). Genome-wide association analysis of seed number per angle in *Brassica napus* L.. Acta Agron. Sin..

[46] Huang Y., Wu T., Cao W., Ma C., Pan S., Xu X. (2020). Evaluation of nutrition and sensory quality of flowering Chinese cabbage based on principal component and cluster analysis (in Chinese with an English abstract). Food Ferment. Ind..

[47] Wang S., Basten C., Gaffney P., Zeng Z. (2007). Windows QTL Cartographer 2.0.

[48] Wang X., Wang H., Long Y., Li D., Yin Y., Tian J., Chen L., Liu L., Zhao W., Zhao Y. (2013). Identification of QTLs associated with oil content in a high-oil *Brassica napus* cultivar and construction of a high-density consensus map for QTLs comparison in *B. napus*. PLoS ONE.

[49] Sun C., Wang B., Wang X., Hu K., Yi B. (2016). Genome-wide association study dissecting the genetic architecture underlying the branch angle trait in rapeseed (*Brassica napus* L.). Sci. Rep..

[50] Sun C., Wang B., Yan L., Hu K., Liu S., Zhou Y., Guan C., Zhang Z., Li J., Zhang J. (2016). Genome-wide association study provides insight into the genetic control of plant height in rapeseed (*Brassica napus* L.). Front. Plant Sci..

[51] Bradbury P.J., Zhang Z., Kroon D.E., Casstevens T.M., Buckler E.S. (2007). Tassel: Software for association mapping of complex traits in diverse samples. Bioinformatics.

[52] Turner S.D. (2014). qqman: An R package for visualizing GWAS results using Q-Q and manhattan plots. bioRxiv.

[53] Tarkowski U.P., Tsirkone V.G., Osipov E.M., Beelen S., Strelkov S.V. (2020). Crystal structure of *Arabidopsis thaliana* neutral invertase 2. Acta Crystallogr. F.

[54] Murakawa M., Shimojima M., Shimomura Y., Kobayashi K., Awai K., Ohta H. (2014). Monogalactosyldiacylglycerol synthesis in the outer envelope membrane of chloroplasts is required for enhanced growth under sucrose supplementation. Front. Plant Sci..

[55] Rottmann T.M., Fritz C., Lauter A., Schneider S., Fischer C., Danzberger N., Dietrich P., Sauer N., Stadler R. (2018). Protoplast-esculin assay as a new method to assay plant sucrose transporters: Characterization of *AtSUC6* and *AtSUC7* sucrose uptake activity in *Arabidopsis* col-0 ecotype. Front. Plant Sci..

[56] Rottmann T., Klebl F., Schneider S., Kischka D., Ruscher D., Sauer N., Stadler R. (2018). Sugar transporter STP7 specificity for l-arabinose and d-xylose contrasts with the typical hexose transporters *STP8* and *STP12*. Plant Physiol..

[57] Keurentjes J.J., Sulpice R., Gibon Y., Steinhauser M.-C., Fu J., Koornneef M., Stitt M., Vreugdenhil D. (2008). Integrative analyses of genetic variation in enzyme activities of primary carbohydrate metabolism reveal distinct modes of regulation in *Arabidopsis thaliana*. Genome Biol..

[58] Oey I., Lille M., Loey A.V., Hendrickx M. (2008). Effect of high-pressure processing on colour, texture and flavour of fruit- and vegetable-based food products: A review. Trends Food Sci. Technol..

[59] Cuerra N., Carrozzi L., Goni M.G., Roura S., Yommi A. (2010). Quality characterization of celery (*Apium graveolens* L.) by plant zones and two harvest dates. J. Food Sci..

[60] Kramchote S., Nakano K., Kanlayanarat S., Ohashi S., Takizawa K., Bai G. (2014). Rapid determination of cabbage quality using visible and near-infrared spectroscopy. LWT-Food Sci. Technol..

[61] Sun J., Li Y., Wu X., Dai C., Chen Y. (2018). SSC prediction of cherry tomatoes based on IRIV-CS-SVR model and near infrared reflectance spectroscopy. J. Food Process Eng..

[62] Li M., Han D., Liu W. (2019). Non-destructive measurement of soluble solids content of three melon cultivars using portable visible/near infrared spectroscopy. Biosyst. Eng..

[63] Raman R., Allen H., Diffey S., Raman H., Martin P., Mckelvie K. (2009). Localisation of quantitative trait loci for quality attributes in a doubled haploid population of wheat (*Triticum aestivum* L.). Genome.

[64] Chun J., Zeng Z. (1995). Multiple trait analysis of genetic mapping for quantitative trait loci. Genetics.

[65] Zhang Z., Ersoz E., Lai C.Q., Todhunter R.J., Tiwari H.K., Gore M.A., Bradbury P.J., Yu J., Arnett D.K., Ordovas J.M. (2010). Mixed linear model approach adapted for genome-wide association studies. Nat. Genet..

[66] Price A.L., Zaitlen N.A., Reich D., Patterson N. (2010). New approaches to population stratification in genome-wide association studies. Nat. Rev. Genet..

[67] Tarka M., Akesson M., Beraldi D., Hernandez-Sanchez J., Hasselquist D., Bensch S., Hansson B. (2010). A strong quantitative trait locus for wing length on chromosome 2 in a wild population of great reed warblers. Proc. R. Soc. B.

[68] Bengt H., Hanna S., Martin S., Maja T., Suvi P., Maria S., Helena W., Dennis H. (2018). Contrasting results from GWAS and QTL mapping on wing length in great reed warblers. Mol. Ecol. Resour..

[69] Yabe S., Hara T., Ueno M., Enoki H., Iwata H. Disagreement between results obtained from GWAS and biparental QTL mapping suggests epistatic interaction. Proceedings of the International Plant and Animal Genome Conference XXII 2014.

[70] Yativ M., Harary I., Wolf S. (2010). Sucrose accumulation in watermelon fruits: Genetic variation and biochemical analysis. J. Plant Physiol..

[71] Winter H., Huber S.C. (2000). Regulation of sucrose metabolism in higher plants: Localization and regulation of activity of key enzymes. Crit. Rev. Biochem. Mol. Biol..

[72] Zhang X., Wang W., Du L., Xie J., Yao Y., Sun G. (2012). Expression patterns, activities and carbohydrate-metabolizing regulation of sucrose phosphate synthase, sucrose synthase and neutral invertase in pineapple fruit during development and ripening. Int. J. Mol. Sci..

[73] Lytovchenko A., Fernie A.R. (2003). Photosynthetic metabolism is severely impaired on the parallel reduction of plastidial and cytosolic isoforms of phosphoglucomutase. Plant Physiol. Biochem..

